# Long-Term Follow-Up of Women with Myalgic Encephalomyelitis/Chronic Fatigue Syndrome (ME/CFS): A 16-Year Longitudinal Study

**DOI:** 10.3390/medicina62061114

**Published:** 2026-06-08

**Authors:** Slavica Tomić, Aleksandra Pastornački, Maja Drljača, Jelena Glogovac, Vanja Bošković, Snežana Brkić

**Affiliations:** 1 Clinic for Infectious Diseases, Clinical Center of Vojvodina, 21000 Novi Sad, Serbia; 2 Medical Faculty, University of Novi Sad, 21137 Novi Sad, Serbia

**Keywords:** ME/CFS, long-term follow-up, cohort study, women, chronic somatic diseases, psychiatric conditions

## Abstract

*Background and Objectives*: Myalgic encephalomyelitis/chronic fatigue syndrome (ME/CFS) is a complex disorder characterized by persistent or relapsing fatigue lasting at least six months, not alleviated by rest and not previously present. It is accompanied by post-exertional symptom exacerbation and non-restorative sleep. Fatigue is often disabling and reduces daily activity by more than 50%. This study aimed to evaluate the long-term frequency of somatic and psychiatric disorders in women previously diagnosed with ME/CFS and to describe the long-term clinical course, laboratory findings, and fatigue-related changes during a 16-year follow-up period. *Materials and Methods*: Sixteen years ago, 40 women diagnosed with ME/CFS according to then-current CDC criteria were enrolled at the Clinic for Infectious Diseases and the Center for Laboratory Medicine, University Clinical Center of Vojvodina. All participants provided informed consent. After 16 years, 20 women agreed to follow-up evaluation. At both time points, participants underwent structured questionnaires, clinical examination, psychological assessment, and comprehensive laboratory testing, including hematological, biochemical, endocrinological, and virological analyses. Fatigue severity was assessed using the FibroFatigue Scale (FFS) and the Multidimensional Assessment of Fatigue (MAF) scale. *Results*: During follow-up, 15% of participants were diagnosed with rheumatoid arthritis, 10% with cervical or breast cancer, 5% experienced premature myocardial infarction, 5% developed bronchial asthma, and 20% were diagnosed with clinical depression. Progression of ME/CFS was observed in 15%, while 5% reported infertility. Additionally, 15% developed arterial hypertension. Only 15% of participants did not report symptom worsening or new diagnoses. *Conclusions*: Over the 16-year follow-up, 85% of women with ME/CFS developed significant somatic or psychiatric conditions. These findings suggest that women diagnosed with ME/CFS may experience substantial long-term somatic and psychiatric disease burden, supporting the need for continued clinical monitoring and individualized follow-up.

## 1. Introduction

The most recent definition of myalgic encephalomyelitis/chronic fatigue syndrome (ME/CFS) was revised in 2025. by the U.S. Center for Disease Control and Prevention (CDC), and according to this definition ME/CFS is a complex clinical entity classified as a chronic and disabling disease that affects multiple organ systems. It is characterized by the presence of mandatory symptoms, the “Major” criteria: an abrupt onset of fatigue that does not resolve with rest and was not present previously, is persistent or relapsing in nature, and lasts for at least six months. This fatigue is marked by exacerbation of symptoms following physical or mental exertion and unrefreshing sleep. Fatigue is continuous, relapsing, and sometimes disabling; it does not subside with rest and reduces average daily activity levels by more than 50%. Prior to the onset of fatigue, the patient was in good health [1,2]. In addition to fatigue, patients present with four or more of the following symptoms (the so-called “Minor” criteria), which occur intermittently over a minimum period of six months and may precede the onset of fatigue: impaired short-term memory and concentration, sore throat, enlarged and tender lymph nodes, joint pain without swelling or redness, muscle pain, and orthostatic hypotension [1,2].

ME/CFS occurs at least six times more frequently in women than in men, although individuals of both sexes may develop the disease [3]. The global incidence ranges between 0.1% and 0.8%. It is estimated that between 17 and 24 million people worldwide, including in the United States, suffer from this condition [4]. The incidence in the Republic of Serbia remains unknown, and the disorder is rarely recognized or formally defined. Diagnosis relies on the presence of characteristic symptoms, functional impairment, and exclusion of other well-defined conditions that could explain the clinical picture, rather than on physical findings or pathological laboratory test results.

Longitudinal studies on ME/CFS are scarce in the literature, although they are essential for clarifying this complex clinical entity. The etiology remains unknown. Most studies suggest that the syndrome is not attributable to a single cause but rather involves multiple etiological factors [2,3,4]. While the precise etiology and definitive treatment remain elusive, there is consensus regarding the role of oxidative stress (OS)—however, it is still unclear whether OS represents a primary cause or a consequence in such a complex system. Research has implicated genetic, infectious, neuroendocrine, immunological, allergic, and psychological factors [3,4,5].

The pathophysiology of ME/CFS is thought to involve interactions between neuroendocrine, humoral, immune, and autonomic nervous system (ANS) dysfunctions, combined with certain psychological predispositions and premorbid personality traits. Any form of stress, whether physical or neurogenic, triggers a rapid and marked increase in adrenocorticotropic hormone (ACTH) secretion, followed within minutes by elevated cortisol release from the adrenal cortex. Analysis of stress responses indicates that activation of the hypothalamic–pituitary–adrenal (HPA) axis during infection or stress induces the release of corticotropin-releasing hormone (CRH) into the central nervous system (CNS) and peripheral tissues [6,7,8]. The CRH system represents the main component in response to stress, with the HPA axis acting as the effector organ. This release contributes to the induction of behavioral responses (cerebral cortex and amygdala), autonomic reactivity (fibers from the locus coeruleus to the brainstem), and hormonal responses mediated via the HPA axis. Today it is hypothesized that due to altered autoregulatory feedback, the organism may transition from hyper- to hyporeactivity of the stress system after a prolonged period of chronic stress, leading to typical symptoms of fatigue, pain, and low mood. The pathogenesis of ME/CFS also involves the interplay between the HPA axis and immune function. Inflammatory mediators such as IL-1 stimulate CRH neurons in the hypothalamus through negative feedback, resulting in immunosuppressive effects of glucocorticoids. If hypothalamic neurons fail to adequately respond to cytokine stimulation, this suppression is absent, leading to a hyperimmune state [6]. The patient’s immune system responds as though faced with persistent infection, regardless of the presence of an actual pathogen. However, during true infection, anti-inflammatory cytokines are produced [5,6]. This dual pattern—hyperreactive immune activity at rest and immunosuppressive responses during infection—also includes mitogenic stimulation of lymphocytes in ME/CFS patients, which is associated with reduced expression of the activation marker CD69 on natural killer (NK) cells. This indicates that despite an initial increase in lymphocyte numbers, the immune response is functionally inefficient due to a predominance of the Th1-mediated immune response [5,6,7,8,9]. Elevated levels of IL-6, IL-1β, and possibly TNF-α at disease onset may contribute to the clinical manifestations of ME/CFS. Depending on the degree of disturbance in the pro-oxidative/antioxidative cellular balance, the biochemical response may result in cellular adaptation and survival, induction of inflammatory and/or immune responses, phenotypic changes, initiation of apoptosis, or necrosis [8,9].

The diagnosis is established by excluding other well-defined clinical entities (ongoing, unresolved, or potential conditions that could explain the fatigue), including psychotic, melancholic, or bipolar depression (but not uncomplicated minor depression), psychotic disorders, schizophrenia, dementia, anorexia or bulimia nervosa, alcohol or substance abuse, and obesity [2,3,4,5,6]. No reliable biological marker currently exists to define ME/CFS. Controversies persist regarding its etiology, biological background, and long-term clinical course. For these reasons, we undertook this study. Women were chosen as the target group because proportionally higher incidence of ME/CFS in this population.

## 2. Materials and Methods

The aims of this study were to assess the long-term prevalence of somatic and psychiatric disorders in a cohort of women previously diagnosed with myalgic encephalomyelitis/chronic fatigue syndrome (ME/CFS), and to describe the long-term clinical course, laboratory findings, and fatigue-related changes observed during the 16-year follow-up period. Additionally, the study aimed to explore possible associations between ME/CFS and the subsequent development of chronic somatic and psychiatric conditions in this cohort.

**Figure 1:**
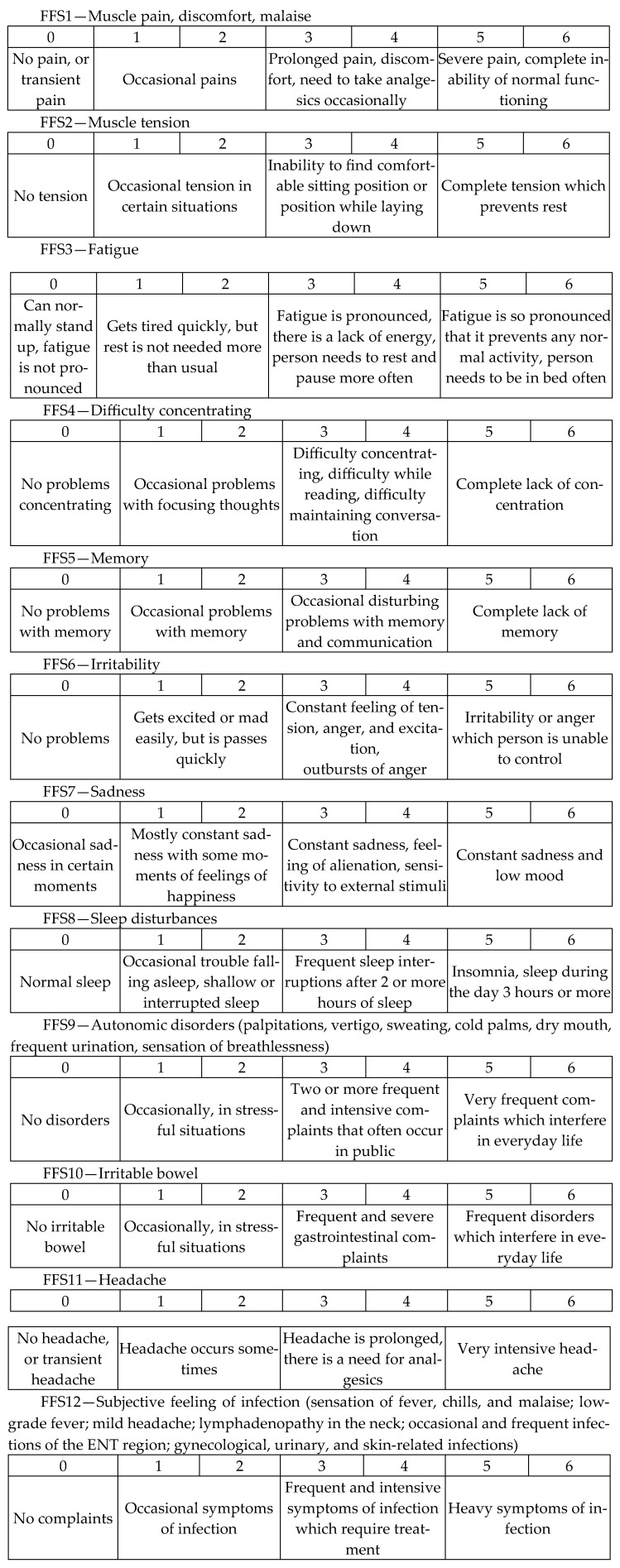
FibroFatigue Scale.

**Figure 2:**
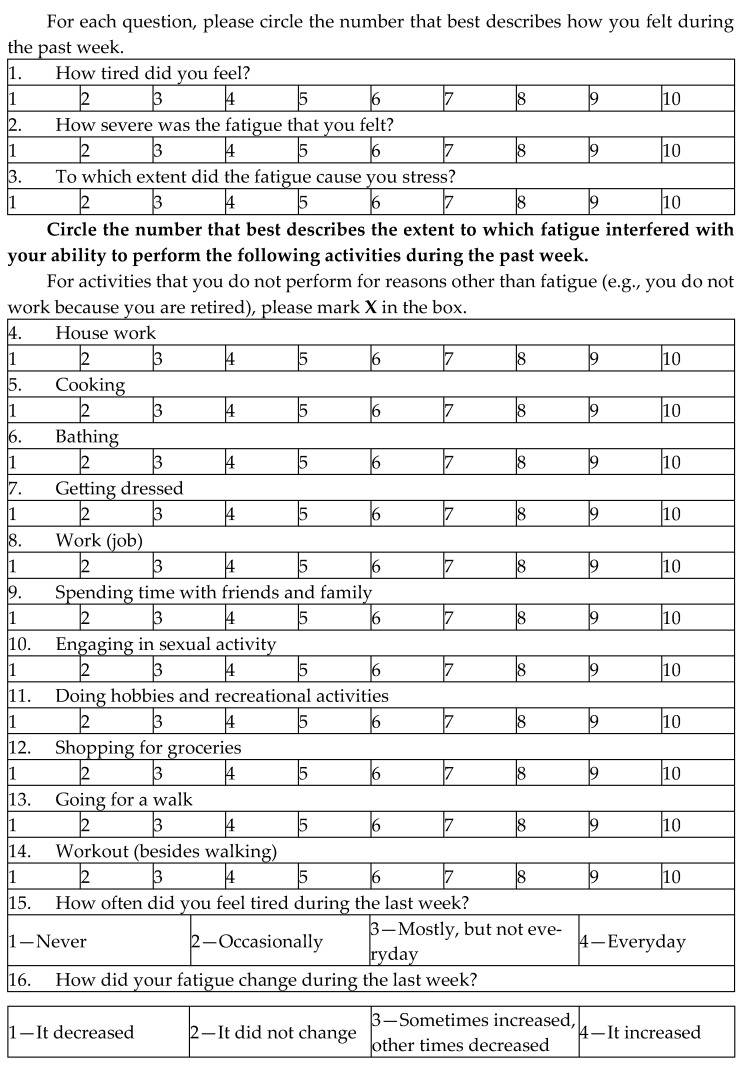
MAF Scale. Instructions: The following questions relate to fatigue and its impact on your activities.

This study was designed as a longitudinal, prospective investigation. Sixteen years ago, we conducted a study involving 40 women with ME/CFS, guided by the diagnostic criteria for ME/CFS available at that time, as recommended by the CDC [1,2,3]. The study was carried out at the Clinic for Infectious Diseases of the University Clinical Center of Vojvodina and the Center for Laboratory Medicine of the same institution. Informed consent was obtained from all 40 participants at baseline. After 16 years, follow-up contact was successfully established with 20 of these patients, while the remaining 20 could not be reached. Some had relocated, a few were unavailable for collaboration, and given the time lapse, their life circumstances had changed. Participants in both assessments underwent evaluation using a structured questionnaire, as well as basic laboratory testing: erythrocyte sedimentation rate (ESR), complete blood count (CBC), routine biochemical urinalysis, fasting blood glucose, liver function tests (AST, ALT, GGT) and renal function tests (urea, creatinine), acute-phase reactants (fibrinogen and C-reactive protein, CRP), serum protein electrophoresis, rheumatoid factor (RF), serum electrolytes (total and ionized calcium, sodium, and potassium), lipid profile (total cholesterol, HDL, LDL, VLDL, non-HDL cholesterol, triglycerides), serum iron levels, endocrine assessment including basal serum concentrations of thyroxine [fT4], triiodothyronine [fT3], thyroid-stimulating hormone [TSH], basal plasma levels of adrenocorticotropic hormone [ACTH], and serum cortisol levels at 08:00 and 18:00. Serological analyses (ELISA IgM and IgG) were performed for Borrelia burgdorferi, cytomegalovirus (CMV), Epstein–Barr virus (EBV), Coxsackie B virus, HBV, HCV, and HIV 1/2. Clinical examination and psychological testing were also performed to exclude psychiatric disorders. At baseline, fatigue was assessed using the FibroFatigue Scale (Figure 1) and the Multidimensional Assessment of Fatigue scale (Figure 2).

FibroFatigue Scale (FFS): Although not intended as a diagnostic tool for ME/CFS, the FFS is a validated instrument for assessing symptom severity and monitoring changes over time, making it suitable for use in clinical trials [7,8,9,10,11]. The FFS is a 12-question, six-degree visual self-assessment scale, administered with investigator support. The total score (summation score) reflects greater fatigue severity and overall symptom burden in ME/CFS. Higher sub-scores for individual items indicate greater symptom intensity. The scale demonstrates high internal consistency (Cronbach’s α = 0.91) and has been validated in previous research [11].

Multidimensional Assessment of Fatigue (MAF) scale is a 16-item self-assessment scale evaluates four dimensions of fatigue [11,12]. All items of this scale refer to the preceding seven days. MAF scale is a self-assessment toll, it is short, but it can clearly differentiate characteristics of subjective pain quantification and its effect on different activities. First dimension of fatigue constitutes intensity of degree of said fatigue which the participants have felt in the aforementioned time period. Second dimension constitutes implies questions about self-assessment of stress caused by fatigue. Third dimension represents intensity of the impact of the fatigue on house work, personal hygiene, work activities, social activities, practicing sports or other recreation activities. Fourth dimension of fatigue constitutes frequency and daily duration of fatigue in the last seven days. The MAF scale provides a Global Fatigue Index (GFI) ranging from 0 (no fatigue) to 50 (severe fatigue). It has demonstrated excellent internal consistency (Cronbach’s α = 0.93) and reliability [9,10,11].

At the 16-year follow-up, the identical diagnostic algorithm was applied. The 20 participants provided written informed consent and willingly attended the reassessment. In addition to the diagnostic algorithm, a detailed medical history was taken, focusing on the participants’ current health status, the development of somatic or psychiatric diseases over the past 16 years, and subjective changes in symptoms. All relevant medical records confirming current diagnoses were reviewed. The study was approved by the Ethics Committee of the University Clinical Center of Vojvodina, approval number 00-407.

Statistical data analysis was performed using the Statistical Package for the Social Sciences (SPSS), version 21. Due to the relatively small sample size and the potential deviation from normal distribution of clinical variables, continuous variables were presented as median values with interquartile ranges (IQR). Comparisons between baseline and follow-up measurements were performed using the Wilcoxon signed-rank test. A *p*-value < 0.05 was considered statistically significant.

## 3. Results

The youngest participant was 40 years old, and the oldest was 63 years old, with a mean age of 51.55 years (Table 1).

Statistical analysis showed that, after 16 years, the patients had statistically significantly lower leukocyte levels (*p* = 0.049), lymphocyte (*p* = 0.035), creatinine levels (*p* = 0.001), free thyroxine fT4 (*p* < 0.001), cortisol levels (*p* = 0.001), IgG CMV (*p* < 0.001) and ACTH levels (*p* = 0.045) compared to baseline. Their erythrocyte levels (*p* = 0.005), AST levels (*p* = 0.006), CRP (*p* = 0.014), C3 (*p* = 0.014), fT3 (*p* < 0.001), total cholesterol (*p* = 0.030) and HDL (*p* = 0.010) were statistically significantly higher than 16 years earlier (Table 2).

Sixteen years after diagnosis, the patients had statistically significantly higher scores on the muscle tension scale (FFS2), with *p* = 0.001; more pronounced concentration problems (FFS4), with *p* = 0.032; poorer memory (FFS5), with *p* = 0.001; and significantly higher anxiety (FFS6), with *p* = 0.022. Problems of intestinal function, such as irritable bowel (FFS10), were significantly more pronounced (*p* = 0.003) and the total FFS fatigue score (FFS) was statistically significantly higher in the patients than 16 years earlier (*p* = 0.002) (Table 3).

The median values of the MAF Global Fatigue Index were 29.7 at baseline and 49.0 at follow-up, while the maximum value remained within the permitted range of the scale. In addition, the Wilcoxon test demonstrated a statistically significant increase in fatigue levels during the follow-up period (*p* < 0.001) (Table 4).

**Figure 3:**
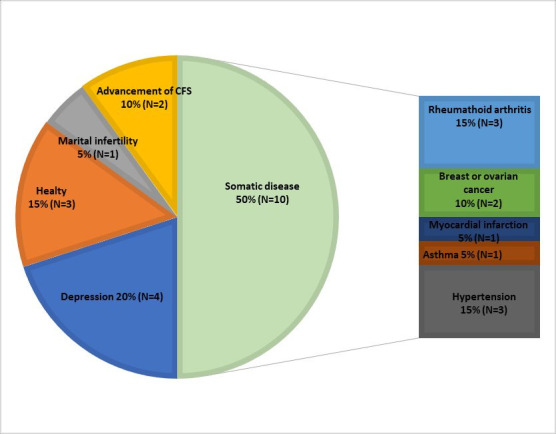
Distribution of somatic and psychiatric diseases and healthy female participants 16 years after the diagnosis of ME/CFS.

Rheumatoid arthritis was present in 15% (N = 3) of participants, some form of cancer in 10% (N = 2), premature myocardial infarction in 5% (N = 1), bronchial asthma in 5% (N = 1), while 15% (N = 3) had developed arterial hypertension. Of all participants, 10% had a marked worsening of chronic fatigue symptoms, 5% (N = 1) had marital infertility, and 15% (N = 3) had no significant complaints. Clinically manifest depression was present in 20% (N = 4) of participants. The results indicate that practically 15% (N = 3) of the participants had no defined systemic disease, whereas 85% (N = 17) developed a defined somatic or a psychiatric disorder (Figure 3).

## 4. Discussion

After 16 years of follow-up, the women originally diagnosed with ME/CFS showed a notable cumulative burden of chronic disease. Before interpreting these findings, two methodological points must be emphasized, as they constrain every conclusion that follows. First, the cohort has aged into a life stage in which most chronic non-communicable diseases become more frequent. The great majority of participants are now postmenopausal, and the contribution of normal biological aging cannot be separated cleanly from any putative influence of ME/CFS. Second, and more importantly, the present study did not include a contemporaneous control group of women without ME/CFS matched for age, geographic region, menopausal status, baseline health, and relevant lifestyle and metabolic risk factors. Without such a comparator, the frequencies of hypertension, depression, malignancy, rheumatoid arthritis, bronchial asthma, and cardiovascular events reported here cannot be judged as higher, lower, or comparable to what would be expected in the general female population of similar age and background over a 16-year period. For this reason, the associations discussed below should be read as descriptive observations within a clinically defined cohort and as a basis for hypothesis generation, not as evidence of elevated risk attributable to ME/CFS.

The decline in the number of participants at the 16-year follow-up was attributable to inability to re-establish contact with previous patients, relocation to another city, and unwillingness to continue participation in the study.

Within these limits, the spectrum of conditions that emerged is nevertheless worth describing. Approximately 85% of participants had developed at least one clinically significant disease by the end of follow-up. Rheumatoid arthritis, malignancy, and bronchial asthma each occurred in a subset of women, arterial hypertension was documented in 20%, clinical depression in approximately 20%, infertility in one woman, and premature myocardial infarction (before the age of 40) in another. ME/CFS symptoms had worsened in 15% of participants, while a further 15% remained free of any newly defined disease. Laboratory follow-up showed mild leukopenia, reduced creatinine concentrations, slightly elevated AST values, and increases in C3 and C4, alongside changes in red cell indices and T3 that are also compatible with relative dehydration in older age. Cholesterol and HDL concentrations had risen, and both cortisol and thyroxine were significantly lower than at baseline. Whether any of these patterns differ meaningfully from age-matched healthy women cannot be answered from the present data; multivariable analysis did not identify a statistically significant baseline variable associated with the later development of organic disease, and we therefore avoid attributing prognostic value to any single laboratory or clinical feature.

The co-occurrence of rheumatoid arthritis in some participants is biologically interesting, even if its frequency in this cohort cannot be compared to a control population. Rheumatoid arthritis and ME/CFS are distinct clinical entities, but several pathophysiological observations have been described in both. Rheumatoid arthritis is characterized by chronic activation of T and B lymphocytes, autoantibody production (RF, anti-CCP), and elevation of pro-inflammatory cytokines such as TNF-α, IL-6, and IL-1β [12,13,14,15]. A subgroup of ME/CFS patients shows a comparable cytokine signature, particularly involving IL-6 and TNF-α, which has been linked to the subjective experience of fatigue [6]. Both conditions are associated with so-called “sickness behavior,” i.e., fatigue, anhedonia, sleep disturbance, and cognitive complaints [13], and both have been linked, with differing degrees of evidence, to central nervous system inflammation, with imaging and biomarker studies in ME/CFS pointing to changes in hypothalamic, thalamic, and limbic regions that may secondarily affect HPA axis activity [13,14,15]. Mitochondrial dysfunction and oxidative stress have been described in muscle and synovial tissue in rheumatoid arthritis and in skeletal muscle in ME/CFS, and may contribute to muscle weakness and exhaustion in both. Clinically, fatigue during rheumatoid arthritis remission can closely resemble ME/CFS, which has prompted speculation about a partial mechanistic overlap rather than a direct causal link. Our data are consistent with such overlap but cannot establish it.

Our earlier work and several independent studies have described neuroendocrine disturbances along the HPA axis in ME/CFS, most often a tendency toward hypocortisolism and disturbed circadian cortisol secretion [9]. In rheumatoid arthritis, acute flares are typically associated with HPA activation, whereas chronic disease may be accompanied by relative adrenal insufficiency, which can itself perpetuate fatigue. Hypotheses framing a subset of ME/CFS as an autoimmune disorder, particularly in patients carrying antibodies to β-2 adrenergic and muscarinic receptors, have been proposed [13,14,15,16]; these remain to be confirmed, and our study was not designed to test them.

A small number of participants developed malignant disease during follow-up. We are cautious about interpreting this finding, since the expected baseline incidence of cancer in postmenopausal women is non-trivial, and without a matched comparator, no statement about excess risk can be made. ME/CFS has been associated with reduced NK-cell cytotoxicity and elevations in IL-1β, IL-6, and TNF-α [3,9,17,18,19]. Chronic low-grade inflammation and oxidative stress can in principle damage DNA and modify cellular metabolism, and impaired immune surveillance could theoretically allow atypical cells to escape elimination. Reactivation of herpesviruses (HHV-6, EBV, CMV) has been documented in some ME/CFS cohorts, and EBV is a recognized oncogenic virus implicated in certain lymphomas, raising the possibility of a virus-mediated pathway between persistent immune activation and hematological malignancy. Mitochondrial dysfunction and oxidative stress in ME/CFS, with reduced ATP synthesis and increased lactate, have been compared with the metabolic phenotype seen in tumor cells (the Warburg effect) [16,17,20], although such analogies should not be overinterpreted. One previous cohort study suggested an increased incidence of non-Hodgkin lymphoma in patients with ME/CFS [17], and a separate analysis reported a higher rate of cancer diagnoses within three months of presentation with unexplained fatigue, particularly in older patients [17]. Cancer-related fatigue and ME/CFS share several biological features, including HPA dysfunction, inflammation, and mitochondrial dysregulation [18,21,22,23,24,25], but the direction of causality, if any, remains unclear. We therefore consider any link between ME/CFS and malignancy in our cohort as a hypothesis worth examining in adequately powered, controlled studies rather than as evidence of a precursor relationship.

Bronchial asthma in some participants is similarly interesting from a mechanistic standpoint. Bronchial hyperreactivity has been reported in a fraction of patients with ME/CFS [19,20], and an immunological profile dominated by Th2 responses has been described in subsets of both conditions. Pro-inflammatory cytokines such as IL-1β, IL-6, and TNF-α are commonly elevated in ME/CFS, and impaired cortisol regulation could in principle aggravate asthma control during exacerbations [9,21]. Autonomic dysregulation, with increased vagal tone and impaired stress responses, has been documented in both disorders, and oxidative stress contributes to airway epithelial injury in asthma [20,21,22]. Shared genetic predispositions (for example, cytokine polymorphisms) and overlapping biomarkers (IL-6, CRP, IgE) have also been described [20,21,22]. Whether these shared mechanisms translate into increased risk of asthma among women with prior ME/CFS cannot be determined here.

Clinical depression was documented in approximately 20% of participants, consistent with studies reporting a high prevalence of affective disorders in ME/CFS, whether as comorbidity or as a consequence of reduced functional capacity and quality of life [18]. Approximately two-thirds of patients with ME/CFS are reported to experience significant psychological symptoms [2,18], and generalized anxiety disorder, panic disorder, and depressive disorders are described as frequent comorbidities. The presence of psychiatric comorbidity does not in itself argue for a purely psychiatric origin of ME/CFS, since depressive disorders themselves involve well-documented disturbances of neurotransmitter balance and neuroinflammatory signaling [4,12,18]. In our cohort, long-term follow-up allowed us to identify several psychological processes that appeared to contribute to depression in individual cases: persistent somatic symptoms with loss of functional autonomy, ruminative cognition, anticipatory anxiety, and the gradual loss of occupational and social roles. Many participants reported that at the start of follow-up, their symptoms had been minimized or dismissed by clinicians; this experience appeared to contribute to internalized stigma and social withdrawal in some patients. Vulnerability factors such as early-life adversity and pre-existing anxious or depressive traits were also evident in retrospective review. Contemporary neuroimmunological models propose shared mechanisms between ME/CFS and depression, including chronic low-grade inflammation, elevated TNF and IL-6, and altered monoaminergic signaling [22]. Reduced central CRH availability has been proposed as one upstream mechanism contributing both to hypocortisolism and to behavioral symptoms such as lethargy, pain, and sleep disturbance in ME/CFS [18,22,23,24,25]. These models are consistent with our clinical observations but, again, cannot be tested in an uncontrolled cohort.

One participant developed marital infertility, defined as failure to conceive after 12 months of regular unprotected intercourse. The relationship between ME/CFS and female infertility has not been clarified, and a single case in our cohort obviously does not support inference of any kind. We note, however, that the literature describes some overlap between the immunological and neuroendocrine features of ME/CFS and those reported in idiopathic infertility, including elevated IL-6 and TNF-α, reduced NK-cell activity, and HPA axis dysregulation [18,26,27]. Mitochondrial dysfunction and oxidative stress have been implicated in oocyte quality, and the psychological burden of infertility (anxiety, depression, somatization) overlaps phenomenologically with ME/CFS [27,28,29]. A retrospective study has reported a lower childbirth rate in the three years preceding a diagnosis of ME/CFS compared with controls [30]. These observations may justify further controlled investigation of reproductive outcomes in women with ME/CFS [31,32,33].

Elevated blood pressure was recorded in 20% of participants, and one woman experienced myocardial infarction before the age of 40. Whether the frequency of hypertension differs from that expected in postmenopausal women of similar background cannot be determined in the absence of a control group, and the single case of premature infarction does not allow any inference about cardiovascular risk attributable to ME/CFS. Mechanistically, oxidative stress with elevated malondialdehyde (MDA) has been described in ME/CFS and has been proposed as a link to early vascular changes [3,26,27,28]. MDA can react with collagen and LDL and may contribute to vascular stiffness independently of classical risk factors [29,31,32,33]. Mitochondrial dysfunction, impairment of respiratory chain complexes I and V, peroxisomal dysfunction, and a prolonged post-exertional oxidative response have all been documented in ME/CFS cohorts [30,34,35], and could plausibly contribute to early endothelial injury. These mechanisms, however, remain a rationale for studying cardiovascular outcomes in controlled ME/CFS cohorts rather than evidence that the present cohort is at higher risk.

A subset of participants showed progression of ME/CFS symptoms, with two women acquiring a formal disability status. Conversely, 15% of women remained free of significant new disease over the 16 years of follow-up. This subgroup may have benefited from a combination of immunological, psychological, and social factors, including a less severe initial illness course, better social support, or undocumented therapeutic interventions; spontaneous improvement of ME/CFS over years has also been described. Among the laboratory variables, the most clinically informative changes were significant reductions in cortisol and thyroxine. These changes are compatible with sustained alteration of the HPA axis, although they are also consistent with normal age-related changes in postmenopausal women and therefore require a matched comparison to be interpreted. The remaining laboratory abnormalities in individual patients were broadly congruent with the chronic conditions they had developed, and the longitudinal medical documentation was used to corroborate clinical events across the follow-up interval.

The FibroFatigue Scale (FFS) showed higher item scores and a significantly increased cumulative score compared with baseline, which is consistent with the parallel emergence of chronic disease, depressive symptoms, and worsening of fatigue in part of the cohort. The Multidimensional Assessment of Fatigue (MAF) scale, which references the preceding seven days and is therefore less subject to long-term recall bias, showed significant increases in fatigue-induced stress, difficulty performing household tasks, and overall fatigue compared with the baseline assessment 16 years earlier.

The principal limitation of the present study is the absence of a contemporaneous, matched control group of women without ME/CFS, which prevents any inference about whether the observed frequencies of somatic and psychiatric conditions exceed those expected in the general population of similar age, geographic region, and menopausal status. In addition, the relatively small final sample size and the 50% loss to follow-up over the 16-year observation period may have introduced selection bias and reduced the statistical power of the study. Since detailed baseline data for participants lost during follow-up were not fully available, sensitivity analyses and direct comparisons between participants who completed follow-up and those who did not could not be performed.

Several of the examined conditions, including myocardial infarction, infertility, malignancy, and bronchial asthma, were observed in only one or a few participants and therefore cannot support broader generalizations or causal interpretations. Furthermore, no a priori power analysis was performed, and the relatively small cohort increases the possibility of type II statistical error. Important potential confounding factors such as menopausal status, body mass index, smoking status, medication use, socioeconomic factors, and baseline comorbidities were not available for multivariable adjustment and may have influenced both clinical and laboratory findings observed during follow-up.

Multivariable analysis did not identify any baseline clinical or laboratory variable significantly associated with the later development of organic disease; therefore, we deliberately avoided interpreting ME/CFS, or any individual clinical or laboratory feature, as a predictor or prodromal stage of subsequent somatic or psychiatric illness. The inflammatory, neuroendocrine, oxidative, autonomic, and mitochondrial mechanisms discussed throughout this section are derived primarily from previous literature and should be interpreted as biologically plausible hypotheses rather than mechanisms demonstrated by the present study.

Within these limitations, our findings provide a descriptive overview of the long-term clinical trajectory of a defined cohort of women diagnosed with ME/CFS and may help guide future adequately powered, prospectively controlled longitudinal studies.

## 5. Conclusions

Taken together, the findings of this 16-year longitudinal study describe a cohort in which the long-term clinical course was rarely benign: 85% of women originally diagnosed with ME/CFS developed at least one clearly defined somatic or psychiatric disorder during follow-up, spanning rheumatoid arthritis, malignancy, bronchial asthma, arterial hypertension, clinical depression, and, in isolated cases, premature myocardial infarction and infertility. The diversity of these outcomes, together with the parallel worsening of fatigue documented on both the FFS and MAF scales, suggests that the trajectory of ME/CFS in middle-aged women extends well beyond the symptom of fatigue itself and intersects with several of the major chronic disease domains of later adult life.

These observations should nonetheless be read with the methodological boundaries of the study clearly in view. In the absence of a contemporaneous control group matched for age, region, menopausal status, and baseline risk profile, the frequencies reported here cannot be compared with the expected occurrence of these conditions in the general female population over an equivalent interval, and no inference can be drawn about excess risk attributable to ME/CFS. Multivariable analysis did not identify any baseline clinical or laboratory variable significantly associated with the later development of organic disease, and for this reason we have deliberately refrained from describing ME/CFS, or any of its features, as a predictor or prodromal stage of subsequent illness. What the data do support is a more modest but clinically relevant conclusion: that women carrying a diagnosis of ME/CFS form a group in which new somatic and psychiatric conditions accumulated steadily over 16 years, and in whom continued clinical attention, periodic reassessment, and individualized follow-up appear well justified, so that emerging disorders can be recognized and addressed at an early stage.

The mechanistic threads running through the conditions observed in this cohort (chronic low-grade inflammation, HPA axis dysregulation, autonomic imbalance, oxidative stress, and mitochondrial dysfunction) are biologically coherent and are echoed in the wider literature on ME/CFS, rheumatoid arthritis, affective disorders, asthma, infertility, and early vascular disease. They do not, in our data, rise to the level of demonstrated pathophysiological links, but they offer a defensible rationale for the next step: adequately powered, prospectively designed studies that include matched controls and a broader panel of candidate biological and psychosocial variables. Such studies will be required to determine whether any of the mechanisms discussed here translate into measurable predictors of long-term outcome, and to clarify the natural history of ME/CFS as these patients move through later decades of life.

## Figures and Tables

**Table 1:** Age distribution 16 years after the initial ME/CFS diagnosis.

N	Minimum	Maximum	Mean
20	40	63	51.55

**Table 2:** Participants’ laboratory tests at the time of ME/CFS diagnosis and 16 years after the initial diagnosis.

	Median	N	IQR	Wilcoxon Test	*p*
Leucocytes	5.865	20	5.078–6.780	2.297	0.049
Leucocytes_0 *	6.695	20	5.170–7.400		
Neutrophils	3.570	20	2.985–4.138	−0.205	0.837
Neutrophils_0	3.490	20	2.835–4.163		
Lymphocytes	1.925	20	1.603–2.145	−2.109	0.035
Lymohocytes_0	2.200	20	1.750–2.620		
Monocytes	0.400	20	0.355–0.483	−1.249	0.212
Monocytes_0	0.435	20	0.385–0.560		
Eosinophils	0.160	20	0.090–0.225	−0.168	0.866
Eosinophils_0	0.125	20	0.090–0.208		
Erythrocytes	4.310	20	4.095–4.585	−2.839	0.005
Erythrocytes_0	4.140	20	3.955–4.390		
Hemoglobin	131.000	20	121.750–138.250	−0.990	0.322
Hemoglobin_0	130.000	20	123.500–132.750		
Thrombocytes	264.500	20	202.500–289.500	−0.821	0.411
Thrombocytes_0	231.500	20	205.000–316.250		
Fibrinogen	3.560	20	2.753–3.983	−1.348	0.178
Fibrinogen_0	3.015	20	2.678–3.760		
ALT	19.000	20	15.000–24.500	−0.910	0.363
ALT_0	18.500	20	16.000–20.000		
AST	21.500	20	19.250–26.500	−2.759	0.006
AST_0	15.000	20	13.000–18.750		
Urea	4.150	20	3.900–5.250	−0.302	0.763
Urea_0	4.450	20	3.475–4.950		
Creatinine	63.000	20	58.750–70.750	−3.363	0.001
Creatinine_0	74.000	20	70.000–86.750		
Potassium	4.200	20	4.000–4.375	−1.047	0.295
Potassium_0	4.155	20	3.963–4.298		
Sodium	140.000	20	138.000–142.000	−0.313	0.754
Sodium_0	141.000	20	139.000–141.750		
CRP	1.650	20	1.000–2.400	−2.457	0.014
CRP_0	0.550	20	0.400–1.278		
C3 complement	1.1450	20	1.048–1.238	−2.110	0.014
C3 complement_0	0.955	20	0.883–1.130		
C4 complement	0.245	20	0.205–0.278	−1.403	0.160
C4 complement_0	0.205	20	0.168–0.275		
T3 hormone	4.450	20	3.950–4.950	−3.9270	<0.001
T3 hormone_0	1.450	20	1.300–1.600		
T4 hormone	11.700	20	11.400–12.275	−3.920	<0.001
T4 hormone_0	90.500	20	82.500–97.300		
TSH	2.160	20	1.070–3.190	−0.672	0.502
TSH_0	2.365	20	1.205–3.275		
Cortisol at 8 h	355.850	20	277.950–451.975	−3.285	0.001
Cortisol at 8 h_0	557.500	20	407.000–627.750		
Cortisol at 18 h	179.700	20	151.725–247.150	−1.437	0.151
Cortisol at 18 h_0	221.500	20	166.000–390.750		
IgG CMV	160.700	20	129.450–194.400	−3.920	<0.001
IgG CMV_0	745.320	20	363.100–1000.000		
ACTH	15.000	20	9.750–20.000	−1.952	0.045
ACTH_0	20.550	20	13.600–35.475		
Triglycerides	0.995	20	0.678–1.285	−1.008	0.313
Triglycerides_0	0.900	20	0.773–1.288		
Cholesterol	6.000	20	5.030–6.565	−2.166	0.030
Cholesterol_0	4.750	20	4.040–6.105		
HDL cholesterol	1.535	20	1.370–1.698	−2.577	0.010
HDL cholesterol_0	1.360	20	1.123–1.480		
LDL cholesterol	3.880	20	3.160–4.445	−4.605	0.108
LDL cholesterol_0	3.220	20	3.000–4.303		

* Laboratory results from sixteen years ago are followed by an underscore and 0. Most recent values are not otherwise marked.

**Table 3:** Values of the FFS in female patients at the time of ME/CFS diagnosis and 16 years after the diagnosis of ME/CFS.

	N	Median	IQR	Minimum	Maximum	Wilcoxon Test	*p*
FFS1	20	2.000	2.000–3.750	1	5	1.670	0.095
FFS1_0 *	20	2.000	2.000–3.000	0	4		
FFS2	20	3.000	2.000–3.000	1	5	3.297	0.001
FFS2_0	20	1.000	0.000–2.000	0	3		
FFS3	20	3.000	3.000–3.000	1	6	1.882	0.060
FFS3_0	20	4.000	3.000–4.000	2	6		
FFS4	20	2.000	2.000–3.750	1	5	2.141	0.032
FFS4_0	20	2.000	1.000–2.000	0	4		
FFS5	20	3.000	2.000–3.000	1	5	3.275	0.001
FFS5_0	20	1.000	0.000–2.000	0	3		
FFS6	20	3.000	2.000–3.000	1	5	2.286	0.022
FFS6_0	20	2.000	1.000–3.000	0	4		
FFS7	20	2.000	0.000–2.000	0	4	1.509	0.131
FFS7_0	20	1.000	0.000–1.750	0	4		
FFS8	20	2.000	2.000–2.750	0	5	1.761	0.078
FFS8_0	20	2.000	1.000–2.000	0	4		
FFS9	20	3.000	3.000–3.000	2	5	1.717	0.086
FFS9_0	20	3.000	2.000–3.750	0	4		
FFS10	20	2.00	1.000–3.000	0	5	2.961	0.003
FFS10_0	20	1.000	0.000–2.000	0	5		
FFS11	20	2.500	2.000–3.000	0	5	0.080	0.936
FFS11_0	20	3.000	1.250–3.750	0	4		
FFS12	20	2.000	1.000–3.750	0	5	0.243	0.808
FFS12_0	20	2.500	2.000–3.000	0	4		
FFS	20	29.500	24.000–33.000	17	54	3.100	0.002
FFS_0	20	23.000	21.000–25.750	12	33		

* FFS—FibroFatigue Scale. Participants’ chosen values for each question from FFS are represented in each row. Values chosen sixteen years ago are followed by underscore and 0. Most recent values are not otherwise marked. Last row represents cumulative score on FFS.

**Table 4:** Values of the MAF scale in female patients at the time of ME/CFS diagnosis and 16 years after the diagnosis of ME/CFS.

	N	Median	IQR	Minimum	Maximum	Wilcoxon Test	*p*
maf_1d	20	9.000	8.000–12.000	7.0	14.0	−2.074	0.038
maf_1d_0 *	20	12.00	10.000–16.000	3.0	18.0		
maf_2d	20	20.000	17.000–20.000	8.0	23.0	−3.828	<0.001
maf_2d_0	20	5.00	3.000–7.000	1.0	9.0		
maf_3d	20	11.500	10.000–13.750	8.0	22.0	−3.824	<0.001
maf_3d0	20	4.500	2.700–7.100	1.4	8.1		
maf_4d	20	6.000	6.000–6.000	4.0	8.0	−3.629	<0.001
maf_4d0	20	15.000	7.500–15.000	2.5	17.5		
maf_gfi	20	49.000	43.250–49.750	39.0	50.0	−3.823	<0.001
maf_gfi0	20	29.700	19.200–38.500	8.9	42.1		

* MAF—Multidimensional Assessment of Fatigue scale. Maf 1 represents participants’ chosen values for questions 1 and 2 on the MAF scale. Maf 2 represents participants’ chosen values for question 3 on the MAF scale. Maf 3 represents participants’ chosen values for question 4 to 14 on the MAF scale. Maf 4 represents participants’ chosen values for questions 15 and 16 on the MAF scale. Most recent values are not otherwise marked.

## Data Availability

The datasets used and/or analyzed during the current study are available from the corresponding author on reasonable request.

## Abbreviations

The following abbreviations are used in this manuscript:

ACTH	Adrenocorticotropic hormone
ALT	Alanine aminotransferase
ANS	Autonomic nervous system
AST	Aspartate aminotransferase
CBC	Complete blood count
CDC	Centers for Disease Control and Prevention
CD69	Cluster of differentiation 69
ME/CFS	Chronic fatigue syndrome
CMV	Cytomegalovirus
CNS	Central nervous system
CRF	Cancer-related fatigue
CRH	Corticotropin-releasing hormone
CRP	C-reactive protein
EBV	Epstein–Barr virus
ELISA	Enzyme-linked immunosorbent assay
ENT	Ear, nose and throat
ESR	Erythrocyte sedimentation rate
FFS	FibroFatigue Scale
FR	Free radicals
GFI	Global Fatigue Index
GGT	Gamma-glutamyl transferase
HBV	Hepatitis B virus
HCV	Hepatitis C virus
HDL	High-density lipoprotein
HIV	Human immunodeficiency virus
HPA	Hypothalamic–pituitary–adrenal (axis)
IL	Interleukin
LDL	Low-density lipoprotein
MAF	Multidimensional Assessment of Fatigue
MDA	Malondialdehyde
ME	Myalgic encephalomyelitis
NK	Natural killer (cells)
OS	Oxidative stress
RA	Rheumatoid arthritis
RF	Rheumatoid factor
SPSS	Statistical Package for the Social Sciences
Th1/Th2	T helper 1/T helper 2 (lymphocyte subset)
TNF	Tumor necrosis factor
TSH	Thyroid-stimulating hormone
US	United States
VLDL	Very low-density lipoprotein
